# Fish nutrient composition: a review of global data from poorly assessed inland and marine species

**DOI:** 10.1017/S1368980020003857

**Published:** 2021-02

**Authors:** Kendra A Byrd, Shakuntala H Thilsted, Kathryn J Fiorella

**Affiliations:** 1 WorldFish, Penang, Malaysia; 2 Master of Public Health Program, Department of Population Medicine and Diagnostic, Cornell University, S2-004 Shurman Hall, Ithaca, NY 14853, USA

**Keywords:** Food security, Nutrition security, Fish access, Small indigenous species, Aquatic food systems, Marine food systems

## Abstract

**Objective:**

Our understanding of the nutrient contribution of fish and other aquatic species to human diets relies on nutrient composition data for a limited number of species. Yet particularly for nutritionally vulnerable aquatic food consumers, consumption includes a wide diversity of species whose nutrient composition data are disparate, poorly compiled or unknown.

**Design:**

To address the gap in understanding fish and other aquatic species’ nutrient composition data, we reviewed the literature with an emphasis on species of fish that are under-represented in global databases. We reviewed 164 articles containing 1370 entries of all available nutrient composition data (e.g. macronutrients, micronutrients and fatty acids) and heavy metals (e.g. Pb and Hg) for 515 species, including both inland and marine species of fish, as well as other aquatic species (e.g. crustaceans, molluscs, etc.) when those species were returned by our searches.

**Results:**

We highlight aquatic species that are particularly high in nutrients of global importance, including Fe, Zn, Ca, vitamin A and docosahexaenoic acid (DHA), and demonstrate that, in many cases, a serving can fill critical nutrient needs for pregnant and lactating women and young children.

**Conclusions:**

By collating the available nutrient composition data on species of fish and other aquatic species, we provide a resource for fisheries and nutrition researchers, experts and practitioners to better understand these critical species and include them in fishery management as well as food-based programmes and policies.

Globally, more than 1 billion people rely on fish for consumption and livelihoods^(1)^. In fish-dependent regions, fisheries provide livelihoods, income and nutritious food. Fish have long been recognised as particularly nutritious, contributing essential fatty acids, micronutrients, such as Fe, Zn, Ca and vitamin A, as well as animal protein^(2,3)^. However, understanding of the nutrient contribution of the world’s wide diversity of fish and other aquatic species remains starkly limited.

The United Nations FAO has catalogued a growing number of fish species, recently expanding data on their nutrient composition. In 2014, a total of 2033 fish species were listed, but nutrient composition is only available for a quarter of these (25·7%, 526 species) in FAO INFOODS, a database commonly used to calculate nutrient consumption^(1,4)^. The nutrient composition of large-sized, marine species of commercial importance is relatively better assessed. In some settings, regional databases provide nutrient composition data; for example, India’s Central Inland Fisheries Research Institute maintains a detailed database^(5)^. Yet in many settings, because of data limitations, fish are often treated as a largely homogenous food group in analysing diets. Innovative modelling approaches have attempted to fill gaps in nutrient composition data, though they too are restricted to ray-finned species by limited data availability^(6–8)^.

The limitation of fish nutrient composition data is particularly problematic because the species consumed by those who are the most food insecure and nutritionally vulnerable are the most poorly accounted. The species caught by small-scale fishers, harvested in inland fisheries and represented by small-sized species (i.e. fish <25 cm at maturity) are largely absent from global databases. A recent study predicting nutrient availability of fish landing sites underscores this point: nutrient content was available for only 17 % of the finfish caught^(8)^. Similarly, a recent report in Nigeria found that nutrient content was available for high-value, large species, while smaller species were largely missing from available nutrient databases^(9)^. The confluence of aquatic biodiversity, nutrition insecurity and high fish dependence necessitates a better understanding of the nutrient composition of species from diverse settings (e.g. across geographies; across inland, marine and aquaculture).

In the few fisheries where nutrient information has been well assessed, findings suggest there are important differences in nutrient composition of different fish species. The nutrient composition of a subset of inland fish in Bangladesh, a country with high fish reliance and diversity, has been well documented^(10–13)^. The study of Bangladeshi inland fish demonstrates high variation within key nutrients across species. For example, in common small indigenous species, Fe per 100 mg of raw, edible parts ranged from 0·46 to 19·0 mg^(13)^. In the same set of fish species, Zn per 100 mg raw, edible parts ranged from 0·60 to 4·7 mg and variations for other nutrients have been reported for both large indigenous fish and introduced fish species^(13)^.

The policy implications of our findings are far reaching as realised and projected fish declines across global species are causing alarm^(1,14)^. Even as incomplete data on nutrient composition hamper our ability to understand the extent and consequences of these declines, fish declines have been highlighted as a particular nutritional concern^(15)^ and for their potential role in meeting the SDG and reducing malnutrition^(16)^. Moreover, evidence from global^(17)^ and national^(18)^ studies shows that better child linear growth is correlated with higher fish consumption. Highlighting fish and other aquatic species that are particularly nutritious will be integral to addressing malnutrition in fish-dependent regions, planning for conservation and management, developing new strategies to promote production of nutritious species and reducing waste and loss of aquatic species.

To address the gap in availability of nutrient composition data on diverse fish species, we reviewed the literature and extracted data on nutrient compositions of species around the world, with an emphasis on small indigenous and other species that are under-represented in global databases. While we focused our search terms on fish, when other aquatic species (e.g. crustaceans, molluscs, animals, etc.) were also analysed, we included these within our review, but did not search for them explicitly. We highlight five nutrients (Fe, Zn, Ca, vitamin A and docosahexaenoic acid (DHA)) that are commonly lacking in the diets of women and young children in low- and middle-income countries and have been analysed in a relatively larger subset of species within our review. Appendix Table A1 provides a review of the highlighted nutrients, their importance and global patterns of their deficiency.

## Methods

We used an iterative process to identify appropriate search terms initially inclusive of a term regarding nutrient composition (food composition, macronutrient composition, micronutrient composition, nutrient composition and nutrition composition) and a term inclusive of fish (small fish, small indigenous fish, micronutrient fish and micronutrient-rich fish). We also piloted use of the term seafood but retained the use of fish due to concerns regarding limiting freshwater species inclusion. We ultimately used the most inclusive terms (fish*, *nutri* composition) and searched three databases (EBSCO Host Agricola, Web of Science and Web of Science using cabicode Food Composition and Quality) and one research journal (Journal of Food Composition and Analysis, search for fish*). To minimise the risk of missing relevant articles, we also searched reference lists of key studies and examined ‘cited by’ references in Web of Science. Searches were conducted through August 2019; no beginning date was applied, and archiving was limited only by the availability of literature online.

We focused our search on fish and retain that terminology throughout. However, when our searches returned nutrient composition analyses of other aquatic animals (e.g. snails and reptiles), molluscs, cephalopods and other shellfish, we included these within the review. The delimitation of ‘fish’ is culturally specific in many instances, with many molluscs, snails and other aquatic species considered fish in some settings, and fish limited to only large body species in others. As we did not search for all types of aquatic species, our representation of them is likely limited.

Inclusion criteria were as follows: articles contained original aquatic species’ nutrient composition analyses of at least one aquatic species for use as human food (as opposed to uses as pet food, livestock feed or aquaculture feed). We defined nutrient composition data as inclusive of macronutrients or micronutrients.

We excluded articles that analysed only large-body marine fish species (e.g. Haddock, Cod and Salmon) for which nutrient composition data are well established; in which nutrient composition values were not reported and could not be obtained from the study author; that relied solely on aquatic food purchased at Western-style supermarkets, rather than local markets, and were unlikely to have been regionally sourced; that focused on how different aquaculture feed alterations affected nutrient composition; or included only data on heavy metal concentrations (e.g. lead and mercury) without also including nutrient data. Even within included studies, we did not include composite products (e.g. complementary foods made with aquatic species) in our review. Unfortunately, reasons for exclusion were not enumerated in the review process, and the number of articles excluded by reason is not available.

Following from our formal inclusion criteria, we note some dimensions of the included studies. As we did not exclude articles based on study type or the laboratory methods used to assess nutrient composition, we remind readers that some analytical methods produce more consistent results and better detect nutrient presence and sub-types of nutrients (such as the multiple forms of vitamin A). Included articles often analysed the same species, and some made comparisons among nutrient composition based on processing method, season and location, in addition to analyses focused on different species allowing for increased understanding of how these differences contribute to nutrient differences.

We examined the paper title and abstract to identify studies that were in English, topically relevant, and may include fish nutrient composition data. Studies were then further screened to ensure they included original fish nutrient composition data and a full text of the manuscript was available. In cases of full texts not being accessible or available for purchase, every effort was made to contact the study author to request a full-text copy and we were successful in obtaining relevant articles in all but three cases.

We did not apply any geographic exclusions. While a number of studies of Chinese species are included in our review, our focus on studies in English did lead to the omission of Chinese references that appeared relevant from their English abstract. Further, our exclusion of articles focused on only large-bodied marine fish species incidentally focused our findings within those regions where diverse species’ nutrient composition has been most analysed.

Nutrient composition data and units were extracted from each study for all macronutrients, micronutrients and heavy metals reported. The fatty acids extracted included alpha-linolenic acid, eicosapentaenoic acid (EPA), DHA, arachidonic acid and linoleic acid, and the protein extraction included separate amino acids. Alpha-linolenic acid, EPA and DHA are omega-3 fatty acids, and are mainly found in fish and seafood. Arachidonic acid and linoleic acid are omega-6 fatty acids, and are commonly found in nuts, seeds, and vegetable oils. We included nutrient composition data for each unique analysis conducted by fish species, processing method, fish location or season when applicable. For the limited subset of studies (*n* 7) that measured replicates of individual fish species harvested in the same conditions (site, season, etc.), the average nutrient value was retained and included as a single entry. Note, given the expense of nutrient analyses, multiple individuals are often combined prior to nutrient composition analysis to create an ‘average’ value.

From within the review, we present a subset of key nutrients and five or more selected species. Micronutrients were selected based on data showing global deficiencies^(19,20)^ and include Fe, Zn, Ca and vitamin A. While additional nutrients, such as vitamin B_12_ or folate, are of global importance, very limited data availability prevented their inclusion. DHA was also selected given that it is an essential fatty acid commonly found in fish and was the most analysed polyunsaturated fatty acid in the collated literature. Additionally, some studies have found low concentrations of DHA in blood within certain regions of Africa^(21)^. Species high in these micronutrients and DHA were selected purposively to demonstrate understudied species that provide these nutrients in settings where there is particular concern about inadequate dietary consumption of these nutrients. In addition, we include nutrient composition data from Atlantic Cod and Atlantic Salmon for comparison. Due to disparities in both laboratory analytical methods and units of measurement, calculating summary data of nutrient composition (e.g. average mg of Fe of marine species) introduces several forms of bias and was not appropriate.

### Contribution of a serving of fish to the recommended nutrient intake

For each of our key nutrients – Fe, Zn, Ca, vitamin A and DHA – we present a calculation where we compare the nutrient content of a given uncooked fish species to the daily recommended nutrient intake (RNI) of women and children at different life stages. These calculations were performed to highlight the variation in fish nutrient composition and density (nutrients per gram). The nutrient composition will fluctuate in response to how the fish are cooked or handled, and other components in the diet (e.g. phytates) influence how much of certain nutrient people absorb; thus, these calculations are not meant to provide individual dietary advice. However, these calculations do allow us to provide an estimate of how certain fish species make potential nutrient contributions to diets. We calculate the percentage of the RNI for pregnant women, lactating women and children aged 6–12 months and aged 12–24 months^(22,23)^ that a serving of fish fulfils. Following previously used methods, in our calculations, we assume a 50 g serving for women and a 25 g serving per d for children^(13)^.

The estimated amounts of Fe, Zn, Ca and vitamin A amounts of each fish are listed in Appendix A2-6. For Fe, we assume 10 % bioavailability^(22)^. The RNI for Fe for pregnant women is based on the WHO 2004 value for women aged 19–50 years, as no specific value for pregnant women is given. The value of 29·4 mg/d is in close alignment with the Institute of Medicine recommendation of 27 mg/d for pregnant women^(21)^. For Zn, we assume moderate bioavailability^(22)^. We calculated Zn requirements by averaging the requirement across the three trimesters of pregnancy and first 12 months of lactation, using a value of 7·5 mg/d for pregnancy and 8·5 mg/d for lactation. The Ca and vitamin A requirements were taken directly from the FAO/WHO 2004 for the ages of children reported, and for pregnant and lactating women.

For DHA, the FAO recommends an intake of 200 mg/d of DHA for pregnant and lactating women and the adequate intake for children 6–23 months is estimated to be 10–12 mg/kg body weight per d^(25)^. Based on the work of Bogard *et al.*^(13)^, we used a figure of 110 mg DHA/d for young children, which is the midpoint of the recommended range of intakes based on the respective body weights of children at 7 months and children at 23 months at the 50th percentile^(25)^. The percentage of the nutrient requirement was based on a 50 g/d serving of fish for pregnant and lactating women, and a 25 g/d serving of fish for children 6–24 months. Exact DHA values and sources can be found in Table A6.

The literature review data are provided in Appendix Table A7.

## Results

### Distribution of nutrient analyses

Our searches yielded 8425 articles, and we ultimately included and reviewed 164 articles analysing the nutrient composition of 1370 entries on fish and other aquatic species (e.g. crustaceans, molluscs; Figure A1). The review includes 515 unique species with multiple species entries analysed across different studies, or across cooking methods, harvest locations or seasons. Fifty percent of species were classified as freshwater and 45 % as marine; 5 % of fish were farmed (65 % of which were freshwater species). Additionally, 14 % of the 515 species were described as small indigenous species (SIS), or a similar term, in at least one study; however, other species might also fit within this categorisation.

**Fig. 1:**
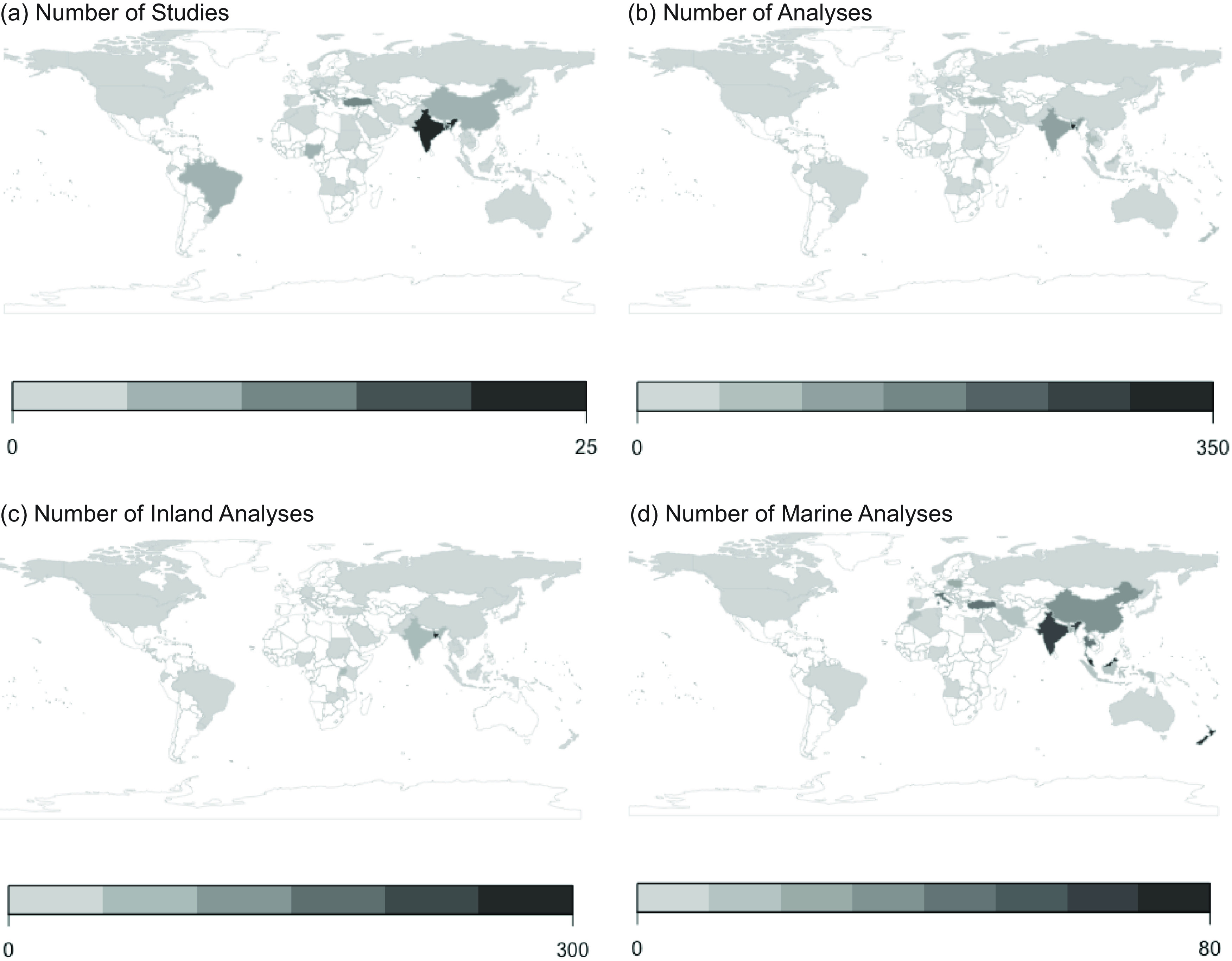
Number of a) studies and b) analyses, as well as c) inland analyses and d) marine analyses. Numbers of analyses refers to the total number of analyses done, which exceeds the number of species analyzed as multiple studies may have analyzed the nutritional composition of the same species, or a study may have compared nutritional composition of the same species across different conditions (e.g., habitats, populations, times of year) resulting in multiple analyses

Studies were conducted in forty-eight countries, with the greatest number of species assessed in South and Southeast Asia (Fig. 1). Analyses of inland species, notably including a wider representation of African species, were conducted in twenty-nine countries, and analyses of marine species were conducted in thirty-four countries (Fig. 1).

### Composition of key nutrients

For each nutrient, we highlight five or more fish and other aquatic species from our review that are high in the given nutrient and provide comparative nutrient data for Atlantic Cod and Atlantic Salmon.

#### Iron

Fe was reported for 535 of the 1370 entries analysed (39·1 %), and the Fe content for the selected species is listed in Table A2. Compared with Atlantic Cod and Salmon, small indigenous fish species and other aquatic species from Bangladesh, India, Laos, and the countries around Lake Victoria (Kenya, Tanzania and Uganda) had a substantially higher Fe content. For example, Jat Punti (*Puntius sophore*), a common small indigenous species in Bangladesh, contains 11·6 mg Fe/100 g of wet weight, compared with 0·38 mg/100 g raw Atlantic Cod (*Gadus morhua* L.) and 0·80 mg/100 g raw Atlantic Salmon (*Salmo salar* L.). Further, the type of Fe (haem *v*. non-haem) found in a food influences bioavailability or the extent to which Fe can be absorbed by the body. In the few cases in which Fe type has been analysed in fish, such as in Mola (*Amblypharyngodon mola*), high concentrations of haem Fe, the more bioavailable type of Fe, have been identified^(26)^.

**Fig. 2:**
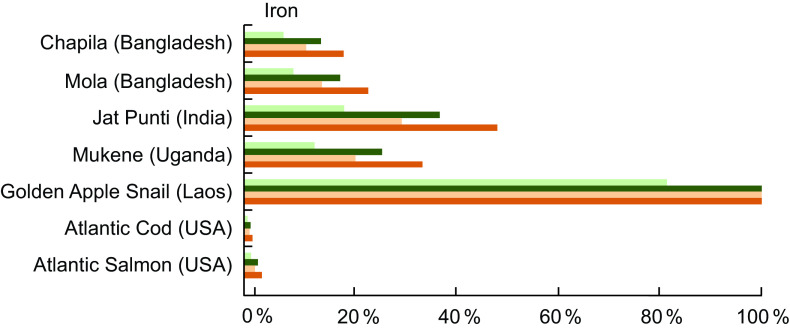
Contribution (%) of recommended nutrient intake (RNI)^(22)^ of iron by fish and other aquatic species. Percentages are estimated based on a 50 g/d serving of fish for pregnant and lactating women, a 25 g/d serving for children 6–24 months, and assuming 10 % bioavailability of iron. The RNI for iron for pregnant women is based on the value of 29·4 mg/d for women aged 19–50 years, as no specific value for pregnant women is given. This is in alignment with the Institute of Medicine (IOM) RDA of 27 mg/d for pregnant women^(24)^. Iron values and sources are given in Table A2. 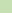, Pregnant women; 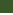, lactating women; 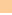, children 6-11 months; 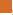, children 12-24 months

High nutrient concentrations of Fe in fish can meet demands for Fe at critical periods in the life cycle. For a lactating woman, a daily serving of Jat Punti fulfils 38 % of her daily Fe needs (Fig. 2). For infants aged 6–11 months, a serving of Jat Punti fulfils 31 % of daily Fe needs (Fig. 2). Other aquatic species also have high Fe composition; the Golden Apple Snail (*Pomacea canaliculata*, de-shelled) from Laos contained 48·0 mg/100 g of wet weight, and a daily serving fully meets the dietary need of lactating women and children 6–24 months of age (Fig. 2).

#### Zinc

Zn was quantified in 31·2 % of entries in our review, and Table A3 lists the Zn content of the selected species. Darkina (Flying barb, *Esomus danricus*) and Mola (*A. mola*) from Bangladesh, Hichiri (Spotty-faced Anchovy, *Stolephorus waitei*) in India and Mukene (Silver Cyprinid, *Rastrineobola argentea*) from the countries around Lake Victoria are four small indigenous species that provide particularly high concentrations of Zn (Table A3). In particular, Hichiri contained 26·0 mg/100 g wet weight of Zn, while our reference fish, Atlantic Salmon and Cod, provided insignificant amounts at <1·0 mg/100 g of raw fish (Table A2). Other aquatic species can also play a role in addressing Zn deficiency, and the Big Apple Snail (Pila sp., de-shelled) provides 12·0 mg/100 g of wet weight (Table A3).

**Fig. 3:**
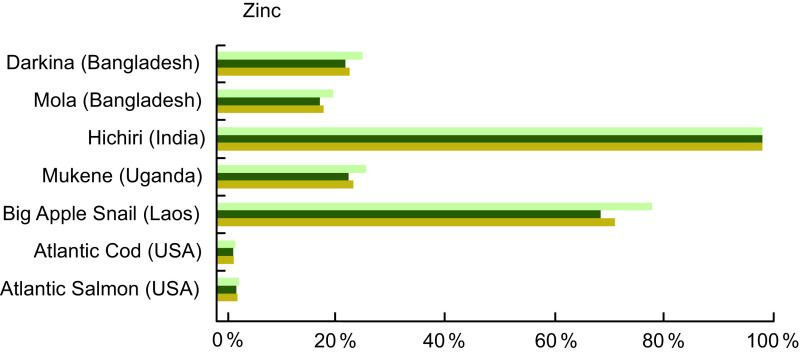
Contribution (%) of recommended nutrient intake (RNI)^(22)^ of zinc by fish and other aquatic species. Percentages are estimated based on a 50 g/d serving of fish for pregnant and lactating women, a 25 g/d serving for children 6–24 months, and assuming moderate bioavailability. For pregnant and lactating women, zinc contributions were calculated by averaging the requirements throughout the three trimesters of pregnancy, and first 12 months of lactation, given that they vary slightly depending on trimester and month of lactation. Zinc values and sources are given in Table A3. 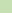, Pregnant women; 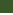, lactating women; 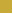, children 6-24 months

Zn is commonly lacking in many diets of low- and middle-income countries^(27)^, and fish high in Zn can address this gap. For example, a serving of Mukene fulfils 27 % of daily recommended Zn intake for a pregnant woman and 24 % for an infant (Fig. 3). A serving of Hichiri from India fulfils over 100 % of the Zn requirement for pregnant and lactating women and children 6–24 months of age, taking into account moderate bioavailability.

#### Calcium

Ca was analysed in 35·0 % of entries in our review, and Table A4 highlights the content from selected species from Bangladesh, India, Laos, Malawi and Uganda. Two small indigenous species from Bangladesh contain high concentrations of Ca, with Jat Punti (Pool barb, *P. sophore*) providing 1711·0 mg/100 g and Kata Phasa (Spined anchovy, *Stolephorus tri*) 1500·0 mg/100 g of raw, edible parts (Table A4). In Malawi, Utaka (*Copadichromis inornatus*) provides 1883·8 mg/100 g of wet weight (Table A4). Notably, again an indigenous snail, the Small Apple Snail (*Cipangopaludina chinensis*, de-shelled) from Laos is also a very good source of Ca, providing 1200·0 mg/100 g wet weight. By comparison, Ca in Atlantic Cod (16·0 mg/100 g raw) and Atlantic Salmon (12·0 mg/100 g raw) is relatively low.

**Fig. 4:**
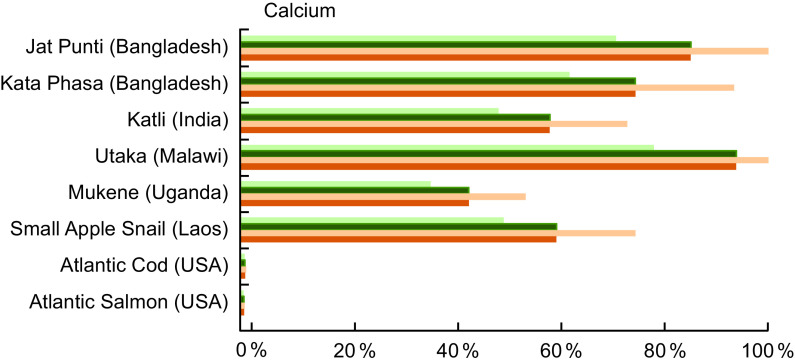
Contribution (%) of mean recommended intake^(22)^ of calcium by fish and other aquatic species. Percentages are estimated based on a 50 g/d serving of fish for pregnant and lactating women, and a 25 g/d serving for children 6–24 months. Calcium values and sources are given in Table A4. 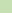, Pregnant women; 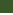, lactating women; 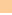, children 6-11 months; 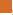, children 12-24 months

Four of the small indigenous species highlighted in Fig. 4 fulfil 50 % or more of the recommended Ca intake for all age categories listed. For example, a serving of Jat Punti fulfils 71 % of the Ca requirement for pregnant woman and 86 % for a lactating woman. For a child aged 6–11 months, a single serving of either Jat Punti or Utaka provides over 100 % of recommended daily Ca intake.

Fish consumed whole, including bones, or as fish powder have high Ca concentrations. These include species that were noted as consumed whole^(28)^ as well as fish by-products that have a low market value and are locally consumed and leftover when fish are processed for an export industry^(29)^. The highest levels of Ca were contributed by species for which ‘plate waste’, leftover after eating, was particularly low, as is typical for small indigenous species compared with moderate- and large-sized fish^(10)^.

#### Vitamin A

Vitamin A was quantified in 18·6 % of the entries analysed in our review, and Table A5 lists the vitamin A content for selected species from Bangladesh, Cambodia and India. In stark contrast to Atlantic Cod and Atlantic Salmon, which both contain 12·0 μgRAE/100 g raw fish, Darkina (*Esomus danricus*), Chanda (*Parambassis baculis*) and Mola (*A. mola*) from Bangladesh all contain over 800 μgRAE/100 g raw, edible parts, with Chanda and Mola containing over 2500 μgRAE/100 g raw, edible parts.

**Fig. 5:**
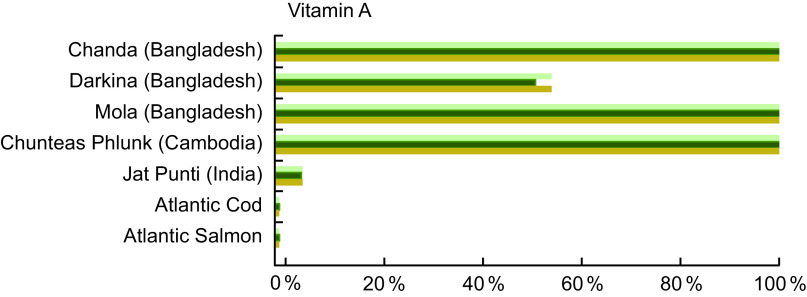
Contribution (%) of recommended nutrient intake (RNI)^(22)^ of vitamin A by fish species. Percentage is estimated based on a 50 g/d serving of fish for pregnant and lactating women, and a 25 g/d serving of fish for children 6–24 months. Vitamin A values and sources are given in Table A5. 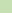, Pregnant women; 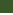, lactating women; 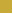, children 6-24 months

Figure 5 shows that vitamin A concentrations in several small indigenous species exceed the recommended intake for vitamin A; thus, a small quantity of these species can make a meaningful impact in meeting vitamin A need. A serving of mola fulfils 157 % and 147 % of the vitamin A recommended intake for pregnant and lactating women, respectively, and fulfils 167 % of the recommended intake for a child 6–24 months of age (Fig. 5). Put another way, to fulfil 100 % of the recommended intake of vitamin A, a pregnant woman would need to consume 29 g, a lactating women 32 g and a child 6–24 months of age 15 g of whole mola. The concentration of vitamin A in mola is particularly high in the eyes and therefore nutritionally advantageous that the fish are consumed whole^(30)^.

#### DHA

**Fig. 6:**
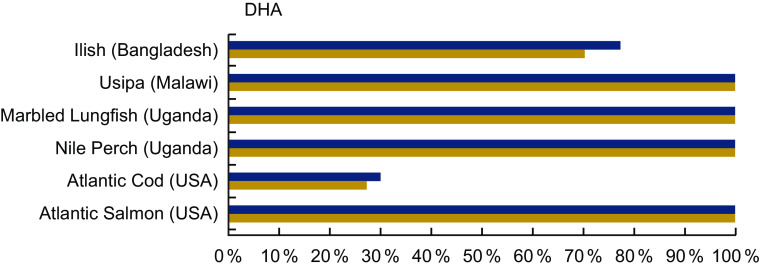
Contribution (%) of daily recommendation of DHA by fish species. The FAO recommends an intake of 200 mg/d of DHA for pregnant and lactating women, and the adequate intake for children 6–23 months is estimated to be 10–12 mg/kg per d^(25)^. Based on the work of Bogard *et al.*^(13)^, we used a figure of 110 mg DHA/d for young children, which is the midpoint of the recommended range of intakes based on the respective body weights of children at 7 months and children at 23 months at the 50th percentile^(26)^. Percentages are estimated based on a 50 g/d serving of fish for pregnant and lactating women, and a 25 g/d serving of fish for children 6–24 months. DHA values and sources are given in Table A6. 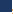, Pregnant and lactating women; 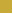, children 6-24 months

DHA was analysed in 33·4 % of the entries in our review, and Table A6 highlights the content from selected species from Bangladesh, Laos, Malawi, and the countries bordering Lake Victoria. Several fish species provide high quantities of DHA. For example, Usipa from Malawi and Nile Perch from Uganda contain 444 mg/100 g wet weight and 970 mg/100 g wet weight. A serving of Atlantic Salmon contains 1115 mg DHA/100 g raw; however, it is not an accessible food to many populations (Table A6). A serving of Usipa, Marbled Lungfish and Nile Perch fulfils over 100 % of the recommended DHA intake for both women and children within the first 1000 d of life, compared with the Atlantic Cod, which provides 30 % or less (Fig. 6).

Notably, freshwater species provide high amounts of DHA in settings where DHA access is of concern. While cold water marine species are often assumed to contribute relatively high levels of essential fatty acids, inland species such as Dagaa or Nile Perch may also make important contributions to dietary DHA, especially where fish are widely and frequently consumed^(31–33)^.

## Discussion

By collating the available nutrient composition data, we provide a resource for fisheries and nutrition researchers, experts and practitioners to better understand the diversity of fish species and include them in programmes and policies. Our findings regarding fish nutrient composition suggest that poorly assessed fish species are high in nutrients of global importance. Such species may be of particular value for meeting the nutritional needs of vulnerable people around the world. Yet, our findings also reveal the geographic limitations in fish nutrient composition data availability, with a subset of fisheries being relatively well assessed, compared with others where data are highly limited.

Many of the fish included within our review offer promising but under-utilised opportunities to increase access to key nutrients and address nutrient deficiencies that cause widespread morbidity and mortality. We present the RNI in our analyses^(22)^. The RNI provide an estimated requirement that ensures the needs of nearly all of a group (97·5 % of the needs of a given age group or life stage) are met and are thus a more conservative estimate than the estimated average requirement (EAR), which provide a nutrient value that meets the needs of 50 % of a group.

### Policy implications

#### Small indigenous fish species and food and nutrition security

Fish are typically treated as a homogenous category in analysing diets. Although ‘fish’ are normally placed on par with poultry, beef or pork, the categorisation of fish refers to thousands of different species which offer unique nutrient profiles to the consumer. While the large marine fish species with better established nutrient composition are unquestionably nutritious, there are relevant distinctions between them and other types of fish, particularly small indigenous species and species that are supplied by global small-scale fisheries.

First, some small indigenous species and other aquatic species caught within small-scale fisheries may be higher in micronutrient content than large, high market value species. For example, the Ca content of Kata Phasa is 93 times higher than that of Atlantic Cod, the Zn content of the Big Apple Snail is 20 times higher than Atlantic Salmon, and the Fe content of Jat Punti is 209 times higher than Atlantic Cod or Atlantic Salmon. Consumption patterns are a factor in these differences. Small fish are commonly consumed whole, leading to a high density and wide variety of nutrients when compared with fish for which only the muscle is consumed^(16)^. For example, Ca delivered by consumption of whole fish is much higher than when fish bones are relegated to plate waste for larger fish species^(30,34)^. Fish for which the head is consumed also deliver higher quantities of micronutrients, particularly vitamin A, often attributed to the consumption of the eyes^(30)^. Further, some fish are rich in Fe of high bioavailability and contain little to no anti-nutrients, which inhibit the absorption of nutrients by the body^(35)^. More detailed accounting of the nutrient contribution of small fish within dietary analyses could better inform the importance of fisheries to nutrition security.

Second, small-sized species are also typically low on the food web, meaning that when heavy metals such as mercury are present, small fish may have relatively lower levels of these heavy metals. Fish body size and trophic level have been associated with methyl mercury in a range of studies^(36,37)^. Both across and within species, larger fish tend to have higher levels of mercury^(38,39)^. Still, fish mercury concentrations are highly variable, and small fish may also dwell in environments where conditions increase mercury methylation^(40)^.

Third, small species and harvests from small-scale fishers are often more financially and physically accessible. Small-scale fishers typically use relatively simple boats and gears to access small indigenous species, compared with the ocean-going vessels and associated gears required to target large marine and pelagic fisheries. The harvest of small indigenous species^(16)^ and inland species^(41,42)^ is also more often directed to local consumption as they are less often exported, typically sold for low prices, available in small quantities, and, some theories suggest, underfished relative to larger fish^(43)^. Further, processing of small species is often easier as they can be dried in the sun or with a small amount of heat, meaning that refrigeration is not required for storage for household use and facilitating transport from rural to urban markets.

Importantly, recognising the nutritional importance of small indigenous and other under-appreciated species is more complex than equating their catch with food and nutrition security. While some communities eat large proportions of the fish they catch, fish remain one of the most widely traded commodities and other communities eat little of their catch or only particular fish types^(44)^. High market value fisheries can contribute substantially to local incomes and thereby food and nutrition security, and a better understanding of local patterns and demographics of sale and consumption are paramount to understanding how and when fisheries can support food and nutrition security^(45)^.

Finally, threats to the availability of small species are looming. Small species are most often targeted for fishmeal and fish oil for use in aquaculture and pet food industries, which may impact their accessibility in the future^(46)^. The expansion of aquaculture has the potential to affect the way small indigenous species are used, their prices and their habitats. Thus, the current and future diversion of these fish to feeds and the effect on food and nutrition security of poor consumers that rely on them should be carefully analysed.

### Nutrient composition data opportunities and challenges

Harmonising and comparing nutrient composition data in fish remains challenging because of differences in units and fish parts measured. The food composition literature uses a range of units (e.g. whole fish, dry weight, muscle and raw, edible parts), of which we suggest raw, edible parts that account for plate waste (e.g. discarded bones) are the most salient metric. Delimiting what is edible, however, must be done carefully. Analyses of muscle tissue may miss substantial edible portions of fish that are widely consumed in many settings, especially for small fish. Conversely, analyses of whole fish may overestimate the nutrients consumed if substantial parts of the fish are discarded. Efforts to take into consideration differences in nutrients as a function of cooking, drying and bioavailability^(10)^ will provide more detailed and relevant data. Some nutrients are also particularly sensitive to analytic discrepancies. For example, vitamin A estimates may be low as vitamin A in fish is found as both 3,4 didehydroretinol (vitamin A_2_) and retinol (vitamin A_1_) with the biological activity of didehydroretinol being 119–127 % higher than retinol^(47)^. Harmonising nutrition composition metrics with a production literature that often uses other units is also a challenge, and a more comparable set of nutrient composition data from fish will provide for improved understanding of the links between fish production and nutrient availability.

In addition to differences reflected by processing and cooking, environmental factors may have substantial effects on nutrient levels in fish. The studies we review highlight the role of harvest location^(48)^ and season^(49–52)^ in affecting nutrient concentrations in fish. For example, for *Mytilus coruscus*, a thick-shelled mussel in China, the concentrations of macronutrients and minerals (e.g. Fe, Mg, Mn and Zn) varied vastly across seasons. In addition, several studies examined different size classes of the same species to assess differences in nutrient composition across fish life spans and found significant differences in amino acids, fatty acids and vitamin A concentrations^(11,53,54)^. Differences across season, location and life stage may reflect important seasonal patterns in temperature and food availability, as well as potential differences across sub-populations of fish and other aquatic species.

Finally, laboratory methods to analyse nutrient composition continue to evolve. The newest methods require state-of-the-art laboratories that are often unavailable in low- and middle-income countries. However, these new techniques have highlighted unique components found in fish. For example, new techniques have shown that washing the fish sample with additional acetone yielded greater haem Fe, and traditional techniques in the past may have underestimated the amount of haem Fe in fish species^(26)^. Further, analyses have often looked only for vitamin A_1_, whereas many small indigenous species are rich in the more bioactive vitamin A_2_^(55)^. While these new methodologies are not part of common laboratory methods to analyse fish nutrient composition, future analytical methods should aim to use these to better assess fish nutrient composition, where possible. Modelling may also prove useful in extending nutrient profiles to better understand nutrient composition of additional species^(6,8)^.

## Conclusions

The cost and laboratory requirements necessary to conduct nutrient composition analyses prohibit analysing the full extent of fish diversity. Still, broader understanding of the nutritional contributions of fish consumed locally and by vulnerable populations, including in the first 1000 d of life, is needed. The incorporation of these species into dietary recommendations and nutrition programmes depends on recognition of their nutrient contributions. So, too, does appreciating the ecosystem services fish and other aquatic species provide, conserving these species, and prioritising local access to them.

Small indigenous species and small-scale fisheries are likely to remain essential for meeting the micronutrient and essential fatty acid needs of the poor. Nutrient-rich fish and other aquatic species could also provide food-based approaches to reducing nutrient deficiencies, with increasing access and consumption offering many advantages over nutrient supplementation, which faces safety and access concerns^(56,57)^. It is the hope of the authors that this review provides as a useful tool for those working around fisheries with poorly characterised nutrient data.

Our review also provides a starting point for future research. Future analyses should examine nutrient patterns across species’ ecological niches, diets or other traits, or how different conditions shape fish nutrient composition as environments change. Future research should also seek to expand modelling of nutrient composition for species that have not been fully analysed and particularly so within geographies where nutritional composition is poorly assessed but fish dependence is high.

## Supplementary material

For supplementary material accompanying this paper visit https://doi.org/10.1017/S1368980020003857. click here to view supplementary material
