# Supplementary material for: Fish nutrient composition: a review of global data from poorly assessed inland and marine species
Source: Public Health Nutr. 2020 Dec 14;24(3):476–86. doi: 10.1017/S1368980020003857 (PMC10195510; doi:10.1017/S1368980020003857)
Supplement: Supplementary file 1 [file S1368980020003857sup.zip › S1368980020003857sup001.docx]

Supplementary Appendix

Figure A1. Study selection diagram of reviewed and included articles.

Table A1. Overview of key nutrients’ biological function, importance, and global deficiency estimates.

| **Nutrient** | **Overview** | **Importance** | **Global Deficiency** |
| --- | --- | --- | --- |
| **Iron** | Iron plays an important role in infant neural development. Iron is necessary for neurotransmitter synthesis^(47, 48)^, development of brain structures in the brain (e.g., dendrites, hippocampus) ^(48)^, and glucose metabolism which fuels neuron growth ^(49)^. Decreased iron reduces erythrocyte concentrations in the blood; anemia occurs when the concentration of erythrocytes fall below a threshold based on age, sex, and physiological status. | Iron-deficiency anemia during pregnancy is associated with adverse delivery outcomes, such as pre-term delivery, low birth weight, and maternal mortality ^(50)^  Young children aged 12 months have the highest iron requirement per kg of body weight compared to other stages of the life cycle ^(51)^. | The WHO estimates that the majority of anemia cases are causes by iron deficiency ^(52)^.  Anemia prevalence in children <2 years is 62% in sub-Saharan Africa and 54% in Southeast Asia. Anemia prevalence in pregnant women is 46% in sub-Saharan Africa and 49% in Southeast Asia. ^(53)^ |
| **Zinc** | Zinc is an important component in the body’s use of macro- and micronutrients and plays a central role in the immune system response ^(20)^. Zinc also plays a key role in protein synthesis indigenous species and growth ^(20)^. | Children who are zinc-deficient are more likely to be stunted and have a reduced ability to fight infection ^(54)^. Thus, adequate zinc intake is especially important during the first 1,000 days of life, from conception through age 2 years. | Many diets globally are predicted to be inadequate in zinc (Beal, Massiot, Arsenault, Smith, & Hijmans, 2017) and because of the role in fighting infection, zinc deficiency is a major public health concern ^(55)^. |
| **Calcium** | Calcium provides rigidity to bones and teeth, is involved in a number of cell signaling processes, and is important for enzymatic and hormonal homeostasis (WHO, 2004). | In pregnant women, low concentrations of circulating calcium can lead to hypertensive disorders, resulting in an increased risk of fetal growth restriction or pre-term birth ^(56)^. | Many people in low- and middle-income countries do not consume sufficient calcium to maintain homeostasis ^(19)^. |
| **Vitamin A** | Vitamin A is essential for growth and development, and plays a key role in the immune system. | Inadequate circulating vitamin A leads to a compromised immune system resulting in an increased risk of mortality, ocular dysfunction, and irreversible blindness in severe cases ^(57)^. | While most global estimates of vitamin A deficiency are outdated ^(58)^, up to 22% of women are estimated to have an inadequate intake of vitamin A ^(59)^.  In 2011, it was estimated that 157,000 deaths of children under five years were attributable to vitamin A deficiency (Black et al., 2013). |
| **DHA (22:6 n-3)** | The importance of DHA and other polyunsaturated fatty acids in early life has become increasingly recognized. | DHA is primarily accessed in utero and through breast milk during the first 1,000 days of life. Studies of DHA in breast milk have found that increased concentrations are associated with children’s cognitive outcomes, visual acuity, and immune response ^(60)^. | Global estimates of DHA intake are varied, but intakes in developing countries are estimated to be below the recommendation, especially during the complementary feeding period ^(61)^. |

Table A2. Selected species of fish and other aquatic animals and their iron content.

| **Fish Species (local name)*** | **Fish Species (common name, scientific name)** | **Study Country** | **Amount of Iron** | **Units**** | **Author(s)** |  |
| --- | --- | --- | --- | --- | --- | --- |
| Chapila | Indian River Shad, *Gudusia chapra* | Bangladesh | 4.55 | mg/100 g fresh weight | Wheal et al, 2016 |  |
| Mola | Mola Carplet, *Amblypharyngodon mola* | Bangladesh | 5.70 | mg/100 g raw, whole fish | Roos et al, 2007 (b) |  |
| Jat Punti | *Puntius sophore* | India | 11.6 | mg/100 g wet weight | Mohanty et al, 2016 |  |
| Mukene or Dagaa | Silver Cyprinid, *Rastrineobola argentea* | Uganda | 8.18 | mg/100 g dried, boiled weight | Kabahenda et al, 2011 |  |
| Golden Apple Snail (de-shelled) | *Pomacea canaliculate* | Laos | 48.0 | mg/100 g wet weight | Nurhasan et al, 2010 |  |
| Atlantic Cod | *Gadus morhua L.* | United States | 0.38 | mg/100 g raw | USDA Database (no. 15015) |  |
| Atlantic Salmon | *Salmo salar L.* | United States | 0.80 | mg/100 g raw | USDA Database (no. 15076) |  |
| *For comparison purposes, two commonly traded fish species, Atlantic Cod and Atlantic Salmon are included.  **Units are those reported in the studies. | | | | | | |

Table A3. Selected species of fish and other aquatic animals and their zinc content.

| **Fish Species (local name)*** | **Fish Species**  **(common name, scientific name)** | **Study Country** | **Amount of Zinc** | **Units**** | **Author(s)** |  |
| --- | --- | --- | --- | --- | --- | --- |
| Darkina | Flying Barb, *Esomus danricus* | Bangladesh | 4.0 | mg/100 g raw, edible parts | Roos et al, 2007 (b) |  |
| Mola | Mola Carplet, *Amblypharyngodon mola* | Bangladesh | 3.2 | mg/100 g raw, edible parts | Roos et al, 2007 (b) |  |
| Hichiri | Spotty-faced Anchovy, *Stolephorus waitei* | India | 26.0 | mg/100 g wet weight | Mohanty et al, 2016 |  |
| Mukene or Dagaa | Silver Cyprinid, *Rastrineobola argentea* | Uganda | 4.1 | mg/100 g fresh weight | Kabahenda et al, 2011 |  |
| Big Apple Snail (de-shelled) | *Pila sp.* | Laos | 12.0 | mg/100 g wet weight | Nurhasan et al, 2010 |  |
| Atlantic Cod | *Gadus morhua L.* | United States | 0.5 | mg/100 g raw | USDA Database (no. 15015) |  |
| Atlantic Salmon | *Salmo salar L.* | United States | 0.6 | mg/100 g raw | USDA Database (no. 15076) |  |
| *For comparison purposes, two commonly traded fish species, Atlantic Cod and Atlantic Salmon are included.  **Units are those reported in the studies. | | | | | | |

Table A4. Selected species of fish and other aquatic animals and their calcium content.

| **Fish Species (local name)*** | **Fish Species (common name, scientific name)** | **Study Country** | **Amount of Calcium** | **Units**** | **Author(s)** |  |
| --- | --- | --- | --- | --- | --- | --- |
| Jat Punti | Pool Barb, *Puntius sophore* | Bangladesh | 1711.0 | mg/100 g raw, edible parts | Roos et al, 2003 (b) |  |
| Kata Phasa | Spined Anchovy, *Stolephorus tri* | Bangladesh | 1500.0 | mg/100 g raw, edible parts | Bogard et al, 2015 (b) |  |
| Katli | Copper Maseer, *Neolissochilus hexagonolepis* | India | 1175.0 | mg/100 g wet weight | Mohanty et al, 2016 |  |
| Utaka | Utaka, *Copadichromis inornatus* | Malawi | 1883.8******* | mg/100 g wet weight (converted from sun-dried weight) | Mumba and Jose, 2005 |  |
| Mukene or Dagaa | Silver Cyprinid, *Rastrineobola argentea* | Uganda | 866.5 | mg/100 g fresh weight | Kabahenda et al. 2011 |  |
| Small Apple Snail (de-shelled) | *Cipangopaludina chinensis* | Laos | 1200.0 | mg/100 g wet weight | Nurhasan et al, 2010 |  |
| Atlantic Cod | *Gadus morhua L.* | United States | 16.0 | mg/100 g raw | USDA Database (no. 15015) |  |
| Atlantic Salmon | *Salmo salar L.* | United States | 12.0 | mg/100 g raw | USDA Database (no. 15076) |  |
| *For comparison purposes, two commonly traded fish species, Atlantic Cod and Atlantic Salmon are included.  ******Units are those reported in the studies.  *******A value of 2693.0 mg/100 g sun-dried fish was reported. Using a moisture content 30.05% for Utaka (Mumba and Jose 2005), we estimated the value for calcium in the wet sample for comparability. | | | | | | |

Table A5. Selected species of fish and their vitamin A content.

| **Fish Species (local name)*** | **Fish Species (common name, scientific name)** | **Study Country** | **Amount of Vitamin A** | **Units of retinol activity equivalent (RAE)** | **Author(s)** | |
| --- | --- | --- | --- | --- | --- | --- |
| Chanda | Indian Glassy Fish, *Parambassis beculis* | Bangladesh | 2510.0 | μgRAE/100 g raw, edible parts | Roos et al, 2002 | |
| Darkina | Flying Barb,  *Esomus danricus* | Bangladesh | 890.0 | μgRAE/100 g raw, edible parts | Roos et al, 2007 (b) | |
| Mola | Mola Carplet, *Amblypharyngodon mola* | Bangladesh | 2680.0 | μgRAE/100g raw, whole fish | Roos et al, 2007 (b) | |
| Chunteas Phlunk | *Parachela siamensis* | Cambodia | 1812.0 | μgRAE/100 g raw, whole, mature fish | Roos et al, 2007 (a) | |
| Jat Punti | Pool Barb,  *Puntius sophore* | India | 86.1*** | μgRAE/100 g wet sample | Mahanty et al, 2014 | |
| Atlantic Cod | *Gadus morhua L.* | United States | 12.0 | μgRAE/100 g raw | USDA Database (no. 15015) | |
| Atlantic Salmon | *Salmo salar L.* | United States | 12.0 | μgRAE/100 g raw | USDA Database (no. 15076) | |
| *For comparison purposes, two commonly traded fish species, Atlantic Cod and Atlantic Salmon are included.  ******Units are those reported in the studies.  ***Reported value in μg/kg wet sample was converted to μg/100 g for comparability. | | | | | |  |

Table A6. Selected species of fish and other aquatic animals and their docosahexaenoic acid (DHA) content.

| **Fish Species (local name)*** | **Fish Species**  **(common name, scientific name)** | **Study Country** | **Amount of DHA** | **Units**** | **Author(s)** |  |
| --- | --- | --- | --- | --- | --- | --- |
| Ilish | Hilsa Shad,  *Tenualosa ilisha* | Bangladesh | 310 | mg/100 g raw, edible parts | Bogard et al, (b) 2015 |  |
| Usipa | Lake Malawi Sardine, *Engraulicypris Engraulicyprisardella* | Malawi | 444*** | mg/100 g wet weight (converted from sun-dried weight) | Yakes-Jimenez et al, 2015 |  |
| Marbled Lungfish | Marbled Lungfish, *Protopterus aethiopicus* | Uganda | 570******** | mg/100 g wet weight | Kwetegyeka et al, 2008 |  |
| Nile Perch | Nile Perch,  *Lates niloticus* | Uganda | 970******** | mg/100 g wet weight | Kwetegyeka et al, 2008 |  |
| Atlantic Cod | *Gadus morhua L.* | United States | 120 | mg/100 g raw | USDA Database (no. 15015) |  |
| Atlantic Salmon | *Salmo salar L.* | United States | 1,115 | mg/100 g raw | USDA Database (no. 15076) |  |
| *For comparison purposes, two commonly traded fish species, Atlantic Cod and Atlantic Salmon are included.  **Units are those reported in studies.  ***The sample was sun-dried and the reported value was 1439 mg/85 g sun-dried fish. Using a moisture content of 72.65% for Usipa (Mumba and Jose 2005), we estimated the value of DHA to be 444 mg/100 g wet weight.  ********Reported values and unit mg/g wet weight were converted to mg/100 g for comparability. | | | | | | |
